# Impact of Warm-Air Withering Methods on Aroma Quality of White Teas from Four Tea Cultivars

**DOI:** 10.3390/foods15122120

**Published:** 2026-06-12

**Authors:** Fan Huang, Yingbo Huang, Xuejiao Gong, Bowen Chen, Juan Zhang, Qian Guo, Wenyi Zhang, Yulong Ye, Zeqiang Ma, Yingchun Wang

**Affiliations:** Tea Research Institute, Sichuan Academy Agricultural Sciences, Chengdu 610066, China; wyc12399@scsaas.cn (F.H.);

**Keywords:** white tea, warm-air withering, volatile components, aroma

## Abstract

While withering is a critical processing step influencing the flavor profile of white teas, the effects of temperature-changing withering remain elusive. This study systematically investigated the variations in two withering methods (natural withering, temperature-changing withering) in volatile compounds of white teas made from four cultivars. The quality of white teas produced from ‘Mingshan No. 131’ (MS131), ‘Fuxuan No. 9’ (FX9), ‘Ziyan’ (ZY), and ‘Fudingdabai’ (FDDB) was evaluated using quantitative descriptive analysis (QDA), headspace solid-phase microextraction coupled with gas chromatography–mass spectrometry (HS-SPME-GC-MS), and odor activity value (OAV) analysis. The sensory evaluation results indicated that temperature-changing withering enhanced the development of sweet and fruity aromas while suppressing grassy notes. A total of 176 volatile compounds were identified, and temperature-changing withering induced significant alterations in the aroma profile, notably increasing the levels of ketones, esters, and alkenes (*p* < 0.05). Based on the criteria of OAV > 1, *p* < 0.05, and a fold change ≥ 1.5 (for upregulated compounds) or ≤0.67 (for down-regulated compounds), key volatile compounds in white teas from the four cultivars were identified. The common upregulated volatile compounds, namely 1-octen-3-one, cedrol, (*E*,*E*)-2,4-heptadienal, and (*E*)-2-hexenal, promoted the fresh flavor profile of white teas. These findings demonstrate that temperature-changing withering optimizes flavor-related metabolites, thereby providing a theoretical foundation for improving white tea processing.

## 1. Introduction

Tea (*Camellia sinensis* L.) is one of the top three non-alcoholic beverages consumed worldwide. Tea is commonly classified into six major categories: white, black, green, yellow, dark, and oolong teas based on the processing methods, sensory qualities, and fermentation levels [1]. Originating from Fujian, China, white tea is also produced in several other Chinese provinces, such as Sichuan, and other countries, including South Korea, Japan, Sri Lanka, and India. White tea is renowned for its natural appearance, delicate flavor, and health-promoting properties [2]. The volatile aroma is an important criterion in the evaluation of tea quality. White tea presents a complex aromatic spectrum, encompassing floral [3], creamy [4], woody [5], aged [6], and cocoa notes [7]. The diversity of these aroma characteristics is shaped by multiple determinants, including tea cultivar, harvesting season, geographical provenance, manufacturing protocols, and duration of post-production storage [8].

The processing of white tea involves two main steps, withering and drying, which contribute to its distinctive quality. The long withering process of 36–72 h is the key step to shaping white tea’s unique quality [9]. The withering methods include outdoor or sunlight withering, charcoal fire withering, tunnel withering, trough withering, indoor withering, tank withering, and combined withering [10]. Previous studies have demonstrated the significant impact of air temperature [11], relative humidity [12], environmental airflow [13], and light [14] on main chemical components, aroma substances, and sensory quality during withering processing. Temperature is a paramount factor influencing the efficacy and efficiency of the withering process in several ways. For instance, the water loss rate, the respiration intensity, and the enzyme activity of withered leaves are closely and positively related to temperature during withering [15,16]. Furthermore, temperature is a major factor in controlling the level of gene transcription, which causes changes in composition and content during withering [17]. Withering is typically done at room temperature between 15 °C and 35 °C. In order to speed up the withering process by increasing cell membrane permeability, frozen withering is also used in trials. It entails putting tea leaves in a deep freezer below 0 °C for a predetermined amount of time [18,19]. Compared with other withering methods, warm-air withering at 35 °C was the most beneficial method for improving Wuyi oolong tea quality [20]. Tan et al. [11] have increased the withering temperature from 25 °C to 40 °C, effectively improving the sensory qualities of Wuyi oolong tea processed from rain-soaked leaves. Elevated temperatures above 38 °C promote the conversion of catechins into theaflavins, a representative pigment of oolong and black teas [21]. During the withering process of black tea, a temperature of 45 °C will cause a decline in flavor quality [22]. Although studies have reported that sunlight withering can elevate *β*-ionone concentration [23] and significantly attenuate the intensity of green aroma [24] of white teas, rainy weather during both spring and autumn often limits the implementation of sunlight withering conditions, which hinders improvements in production efficiency. Therefore, our research focus remains on indoor withering. Based on these literature reports, we propose a variable temperature withering technique. During the long-term withering process of white tea, incorporating a short-term warm-air (50–60 °C) withering treatment can improve tea quality. However, the impact of changing withering temperature technology on the aroma of white tea has not yet been explored.

In addition to the withering process, different tea cultivars also affect the formation of tea aroma. In addition to conventional cultivated tea varieties of white tea, such as Fuding Dabaicha, Fuding Dahaocha, and Fuan Dabaicha, researchers have evidenced that more tea cultivars have the potential to develop specific white teas [25]. Mingshan No.131 and Fuxuan No.9, as local tea cultivars in Sichuan, are famous for their finished-product black teas [26] and green teas [27]. ‘Ziyan’ (*Camellia sinensis* L. Kuntze) is an anthocyanin-rich tea cultivar in recent years, and the anthocyanin content of fresh leaves (one bud and two leaves) in spring is 0.88 mg·g^−1^ [28]. Due to the high anthocyanin contents of ‘Ziyan’ tea, studies have been conducted on food processing, such as tea wine [29], nutraceutical pigment, and other natural foods [30]. The volatile components of Fuding Dabaicha were well-established; however, there were rare studies on white teas produced from fresh leaves of Mingshan No. 131, Ziyan, and Fuxuan No. 9.

In this study, the most widely planted tea cultivars in Sichuan, namely, ‘Mingshan No. 131’ (MS131), ‘Fuxuan No. 9’ (FX9), ‘Ziyan’ (ZY), and ‘Fudingdabai’ (FDDB), were employed to experiment with the withering process of white tea. The technology of temperature-changing withering was employed to influence the sensory quality and flavor profile evolution of white tea by analyzing variations in volatile compounds through quantitative descriptive analysis (QDA), headspace solid-phase microextraction coupled with gas chromatography–mass spectrometry (HS-SPME-GC-MS). Although warm-air withering has been reported, this study is novel in systematically comparing conventional and temperature-changing warm-air withering across four distinct cultivars and identifying common key odor-active volatiles that drive aroma quality differences.

## 2. Materials and Methods

### 2.1. Tea Samples Treatment

**Figure 1:**
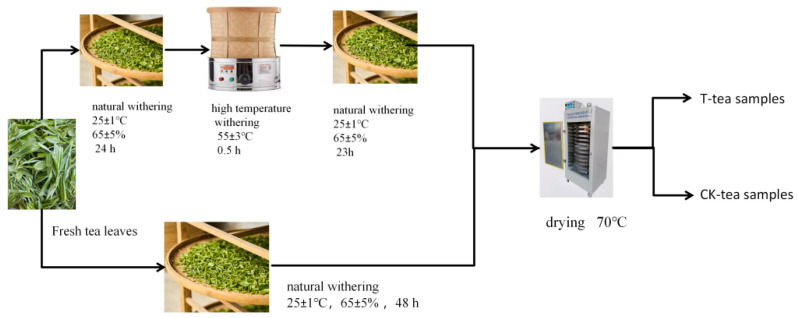
Production process of different tea samples treatment.

Fresh tea leaves (one bud with two leaves, cultivar Mingshan No. 131, Fuxuan No. 9, Ziyan, and Fudingdabai) were harvested in May 2024 from the Wenjun Tea Garden in Wolong County, Chengdu City, Sichuan Province, China. The detailed processing steps and parameters are as follows (Figure 1): (1) For the CK samples, all fresh leaves were evenly spread in the wooden water sieves (diameter: 100 cm, area: 0.785 m^2^), with 1.5 kg per sieve and an initial thickness of 1.5 ± 0.3 cm (measured at five random points), and subjected to natural withering under ventilated conditions at room temperature (25 ± 1 °C) and 65 ± 5% relative humidity for 48 h. (2) For the T (temperature-changing withering) samples, all fresh leaves were evenly spread in the wooden water sieves (diameter: 100 cm, area: 0.785 m^2^), with 1.5 kg per sieve and an initial thickness of 1.5 ± 0.3 cm, and subjected to natural withering under ventilated conditions at room temperature (25 ± 1 °C) and 65 ± 5% relative humidity for 24 h. Next, maintained at a high temperature of 55 ± 3 °C for 0.5 h in the baking machine. Next, maintained at a temperature of 25 ± 1 °C and a relative humidity of 65 ± 5% during withering for 23 h. (3) Final drying, all treated tea leaves were dried uniformly in a dryer at 70 °C until the moisture content met the finished tea requirements. (4) The wooden water sieves were placed on the tea withering racks. The withering racks were positioned away from direct airflow. (5) The indoor temperature and humidity were regulated using an air conditioner and a humidifier, while adequate ventilation was maintained within the room.

All samples (FX9-CK, FX9-T, MS131-CK, MS131-T, ZY-CK, ZY-T, FDDB-CK, FDDB-T) were repeated four times each, which were processed independently, respectively.

### 2.2. Sensory Evaluation Merged with Quantitative Descriptive Analysis (QDA)

We utilized QDA to illustrate the odor profiles of tea samples in this Research. Refer to GB/T 23776-2018 [31], titled “Tea Sensory Evaluation Methods”. A quantity of three grams of tea was placed into a 150 mL cup, followed by the addition of boiling water for a duration of 5 min to assess the aroma and taste. The evaluation panel included five professionals involved in tea production and sensory evaluation, consisting of three males and two females. The reviewers’ ages ranged from 23 to 53 years, and they all held professional tea sensory evaluation certificates. For this study, they were trained using identical tea reference standards for three preliminary sessions and three replicated evaluation sessions. To express the flavor of white teas, five attributes were employed: pekoe, sweet, fresh, floral, and fruity. Using an 11-point rating system that went from 0 (nonexistent) to 10 (very intense), panelists assessed each attribute’s intensity. The radar chart was made using the data after the average scores for each attribute were gathered. Protocols were upheld throughout the study to protect each participant’s rights and privacy. Every member of the group was informed prior to the experiment, and each participant provided signed informed consent.

### 2.3. Analysis of Volatile Metabolites

For white tea samples, 5 g was taken and ground into tea powder according to the Chinese standard (GB/T 8303-2013 [32]). A sample vial was filled with precisely 5.00 g of finely powdered tea sample powder. Following the addition of 10 μL of ethyl decanoate (100 mg·L^−1^) as the internal standard, 50 mL of boiling deionized water was subsequently added. To achieve pre-equilibration, the vial was sealed right away and put in a thermostatic water bath at 60 °C with stirring for five minutes. The temperature was kept at 60 °C during the extraction process, and the DVB/CAR/PDM-coated fiber (50/30 μm; 1 cm; Supelco, Inc., Bellefonte, PA, USA) was then placed into the headspace above the tea infusion and exposed for 50 min to adsorb volatile chemicals. In order to analyze aroma chemicals, the fiber was finally removed and put into the GC-MS injection port. For GC-MS analysis, the volatile chemicals were finally desorbed for three minutes at 240 °C.

A GC system (7890A, Agilent Technologies, Santa Clara, CA, USA) and an MS detector (5975C, Agilent Technologies, Santa Clara, CA, USA) were used to perform the GC-MS analysis. The carrier gas was helium (99.999%) with a steady flow of 1.0 mL·min^−1^, and the column was an HP-5MS type (30 m × 250 μm × 0.25 μm, Agilent Technologies; Santa Clara, CA, USA). The following were the methods used to raise the GC temperature: After holding at 50 °C for five minutes, the temperature was gradually raised to 180 °C at 3 °C·min^−1^, and then it was raised to 250 °C at 10 °C·min^−1^ and held for three minutes. The splitless injection mode was used. The following were the MS conditions: The quadrupole temperature was 150 °C, the ion source temperature was 230 °C, the ionization mode was EI, and the mass scan scope was 50–550 m/z; −70 eV was the electron impact ionization.

The initial identification of volatiles was carried out through automatic matching by comparing the mass spectra via the software, and the compound with the highest matching degree was then listed as the potential correct compound. As a quality control criterion, only compounds with an average MS similarity criterion of >80% were retained. Next, the retention index (RI) of volatile compounds was automatically calculated using a series of n-alkanes (C_7_–C_40_, J&K Scientific Corporation, Beijing, China). Volatiles with an RI difference of less than 20 between the experimental and reference values were retained for further analysis. The mass spectrometer (5975C, Agilent Technologies, Santa Clara, CA, USA) operated in selected ion monitoring (SIM) mode for analyte identification and quantification. Volatile components were identified by comparing the obtained mass spectra with reference mass spectra from the National Institute of Standards and Technology (NIST) mass spectral library (V 14.L). The concentrations of volatile compounds were calculated using the following formula:

C*_x_* = C*_is_* × (PA*_x_*/PA*_IS_*)

where *C_x_* is the concentration of any volatile compound (µg·L^−1^); *C_is_* is the mass concentration of the internal standard (µg·L^−1^); *PA_x_* is the peak area of any volatile compound; *PA_IS_* is the peak area of the internal standard.

### 2.4. Calculation of Odor Activity Values (OAVs)

The OAVs of volatile compounds were calculated according to the formula OAV = C/OT, in which C is the concentration of a volatile compound in tea soup, and OT is its odor threshold in water. The odor thresholds of volatile compounds were obtained from https://www.odour.org.uk/odour/index.html (accessed on 1 January 2026) and previous papers. Volatile compounds with an OAV > 1 are generally regarded as aroma-active constituents that contribute to the overall flavor profile of tea [33].

### 2.5. Statistical Analysis

The figures, radar diagrams, histograms, and pie charts were generated with the Origin software 2024. The Venn diagram, the significant differences were calculated by one-way analysis of variance (ANOVA), and principal component analysis (PCA), volcano plots and bar charts were performed by the Metware Cloud online platform (https://cloud.metware.cn, accessed on 1 January 2026).

## 3. Results and Discussion

### 3.1. Sensory Quality Analysis

**Figure 2:**
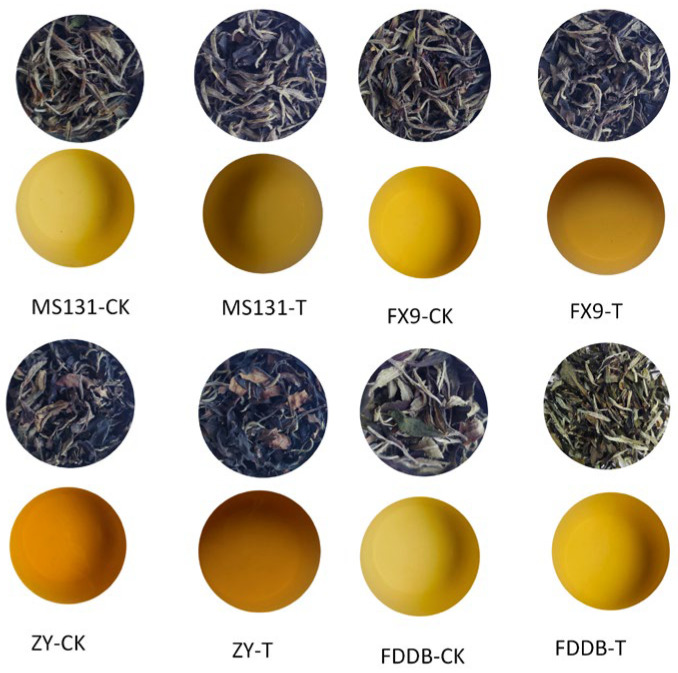
Representative appearance and infusion of eight samples from four tea cultivars.

**Figure 3:**
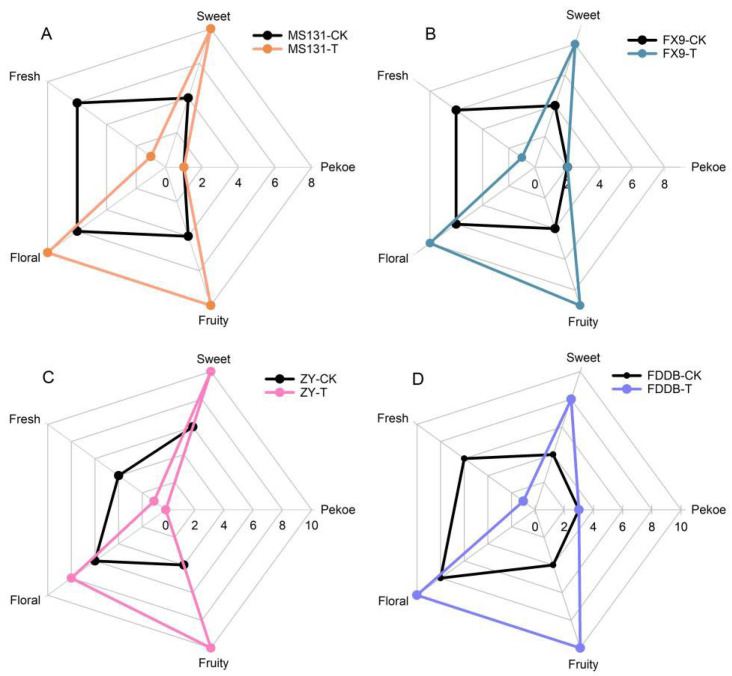
Sensory evaluation merged with quantitative descriptive analysis (QDA) of eight tea samples on five representative aroma, including pekoe, sweet, fresh, floral, and fruity aroma. (**A**) MS131-CK and MS131-T, (**B**) FX9-CK and FX9-T, (**C**) ZY-CK and ZY-T (**D**) FDDB-CK and FDDB-T.

Although white teas are considered the least processed tea, withering methods are highly associated with sensory attributes. Representative appearance, infusion, and leaf base of eight samples from four tea cultivars are shown in Figure 2. It was very obvious that white teas of temperature-changing withering were easily distinguished by their appearance and infusion. Then, sensory evaluation was used to select the aroma characteristics of white teas processed by temperature-changing withering. Five representative aroma attributes were recorded, including pekoe aroma, sweet aroma, fresh aroma, floral aroma, and fruity aroma, and the results were plotted in Figure 3. Four radar charts have presented the aroma profiles of four tea cultivars across various dimensions (Figure 3). For CK samples, fresh and floral aromas are premium attributes in FX9-CK, MS131-CK, and FDDB-CK, and sweet and floral aromas are premium attributes in ZY-CK. For T samples, sweet, floral, and fruity aromas are premium attributes in FX9-T, MS131-T, ZY-T, and FDDB-T. Sweet and fruity aroma scores were significantly elevated in all T samples compared to all CK samples. In addition, floral aroma scores were elevated after temperature-changing withering. Inversely, fresh aroma scores were significantly decreased in all T samples compared to all CK samples. Critically, all temperature-changing withering suppressed grass aroma while enhancing desirable attributes. These results indicate that temperature-changing withering enhances sweet and fruity aroma development of white teas.

### 3.2. Overall Situation of Aroma Composition

#### 3.2.1. The Quantity of Aroma Substances

**Figure 4:**
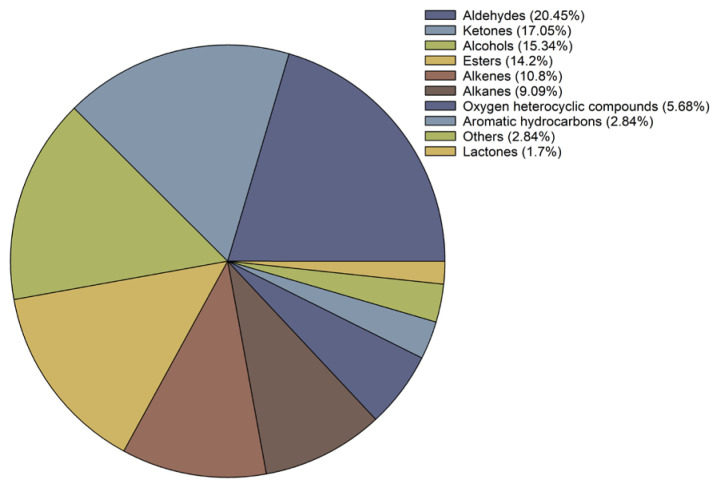
Pie chart with the number of volatiles in all samples.

Conducting both qualitative and quantitative assessments of volatile compounds is essential for evaluating the overall quality of white tea [10]. The unique aroma profile of white tea is intrinsically tied to its endogenous volatile compound synthesis, where the proportions of various volatile components shape its special characteristics [34]. In the analysis of all tea samples, a total of 176 volatile compounds were identified (Figure 4), categorized into 27 alcohols, 36 aldehydes, 16 alkanes, 19 alkenes, 5 aromatic hydrocarbons, 25 esters, 30 ketones, 3 lactones, 10 oxygen heterocyclic compounds, and 5 others. In MS131-CK vs. MS131-T, FX9-CK vs. FX9-T, ZY-CK vs. ZY-T, and FDDB-CK vs. FDDB-T, the number of volatile compounds was 160, 160, 165, 165, 164, 164, 163, and 163 in eight samples.

#### 3.2.2. The Content and Percentage of Volatile Compounds

**Figure 5:**
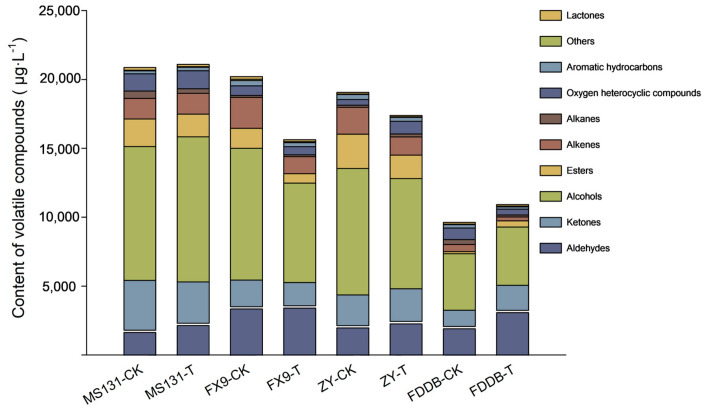
The contents of the volatile category from eight tea samples (μg·L^−1^).

**Figure 6:**
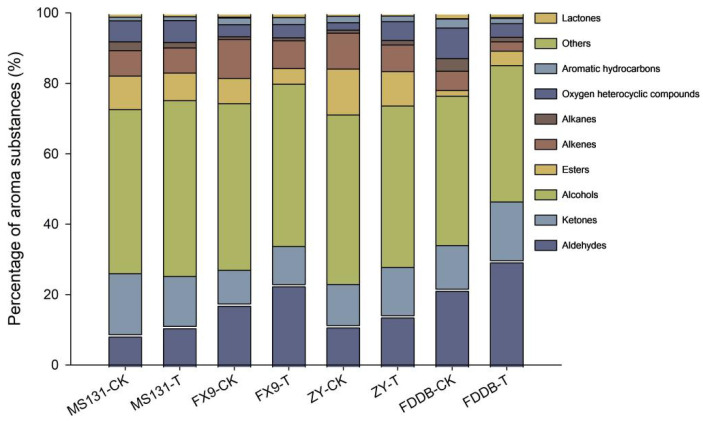
The influence of the new withering process on the proportion (%) of aroma substances in four varieties of white tea.

To investigate the differential changes in aroma after temperature-changing withering, the volatile compounds of four tea cultivars were comparatively analyzed. The contents of the volatile category are shown in Figure 5, and the percentages of the volatile category are shown in Figure 6. Through temperature-changing withering on MS131, there was no significant difference (*p* > 0.05) in total contents of volatile compounds between MS131-CK (20,870.796 μg·L^−1^) and MS131-T (21,094.933 μg·L^−1^). In MS131 samples, the primary volatile compounds were alcohols, which were notable for their floral, sweet, and fruity olfactory profiles. There was no significant difference (*p* < 0.05) in total contents of alcohols between MS131-CK (9720.149 μg·L^−1^) and MS131-T (10,535.795 μg·L^−1^). However, the proportion of alcohols in MS131-CK (49.905%) was increased significantly (*p* < 0.05) compared to MS131-CK (46.558%). Alcohols in tea, including straight and branched chain alcohols from Strecker’s aldehyde reduction or the hydrolysis of glycoside precursors, enhance the variety of tea aroma and significantly impact the overall aroma quality of tea. Compared with MS131-CK, the contents of aldehydes and aromatic hydrocarbons were significantly enhanced in MS131-T (*p* < 0.05), and the proportions of aldehydes and aromatic hydrocarbons also conformed to this phenomenon. Previous studies have demonstrated that most of the aldehydes were positively correlated with the green and fresh flavor of tea broth [35]; aromatic hydrocarbons, with floral and fruity aromas. Conversely, the contents and the proportions of ketones, esters, alkanes, lactones, and others in MS131-T were decreased compared with MS131-CK (*p* < 0.05).

After temperature-changing withering on FX9, the contents of total volatile compounds were decreased significantly (*p* < 0.05) in FX9-T (15,633.338 μg·L^−1^) compared with FX9-CK (20,212.519 μg·L^−1^). And the contents of ketones, alcohols, esters, alkenes, aromatic hydrocarbons, lactones, and others were decreased significantly (*p* < 0.05) in FX9-T compared with FX9-CK. Contrary to the above-mentioned changes in contents, the proportions of alcohols, aromatic hydrocarbons, and lactones have no significant difference (*p* > 0.05) after temperature-changing withering. Ketone compounds and aldehyde compounds contribute to the fruity and fresh aroma profiles. The proportions of aldehydes and ketones were enhanced significantly in FX9-T compared with FX9-CK (*p* < 0.05), suggesting that temperature-changing withering treatments favor traditional withering.

After temperature-changing withering on ZY, the contents of total volatile compounds were decreased significantly in ZY-T (17,390.816 μg·L^−1^) compared with ZY-CK (19,072.675 μg·L^−1^). Only the contents of alkanes and aldehydes in ZY-T (215.106 μg/L, 2430.679 μg·L^−1^) were enhanced significantly (*p* < 0.05) compared with ZY-CK (156.407 μg·L^−1^, 2129.090 μg·L^−1^), and the proportions of alkanes also conformed to this phenomenon. On the contrary, the contents of esters, alkenes, aromatic hydrocarbons, and others were decreased significantly (*p* < 0.05) in ZY-T compared with ZY-CK, and the proportions also conformed to this phenomenon. After temperature-changing withering on FDDB, there was no significant difference (*p* > 0.05) in total contents of volatile compounds between FDDB-CK and FDDB-T. And the contents of aldehydes, ketones, esters, and others were enhanced significantly (*p* < 0.05) in FDDB-T compared with FDDB-CK, and conversely, alkenes, alkanes, oxygen heterocyclic compounds, and aromatic hydrocarbons in FDDB-T were decreased significantly (*p* < 0.05) compared with FDDB-CK. The changes in the proportion of the above volatile categories were consistent with the change in contents. Notably, the proportion of alcohols and lactones in FDDB-T was significantly enhanced compared with FDDB-CK (*p* < 0.05), but the contents of substances did not change significantly (*p* > 0.05).

Comprehensively, after temperature-changing withering, the proportions of aldehydes were enhanced significantly (*p* < 0.05) in white tea samples of four tea cultivars. This common change is likely attributable to the short-term high-temperature treatment during the withering process, thereby leading to a significant increase in the proportion of aldehydes among the aroma compounds. The proportions of ketones were enhanced significantly (*p* < 0.05) in FX9-T, ZY-T, and FDDB-T, compared with FX9-CK, ZY-CK, and FDDB-CK. Esters, known for their sweet, fruity, and floral notes, significantly contribute to tea aroma quality [35,36,37]. The proportions of esters were decreased significantly (*p* < 0.05) in MS131-T, FX9-T, and ZY-T, compared with MS131-CK, FX9-CK, and ZY-CK. The proportions of alkenes were decreased significantly (*p* < 0.05) in FX9-T, ZY-T, and FDDB-T, compared with FX9-CK, ZY-CK, and FDDB-CK.

Collectively, these cultivar-specific responses demonstrate that temperature-changing withering does not uniformly alter volatile profiles but rather interacts with intrinsic cultivar characteristics to reshape aroma chemistry. The consistent increase in aldehyde proportions across all four cultivars points to a shared mechanism involving accelerated lipid oxidation coupled with the degradation of alcohols, amino acids, and unsaturated fatty acids, serving as a metabolic hallmark of this processing innovation. These findings provide a theoretical basis for cultivar-specific optimization of withering protocols in white tea manufacturing.

#### 3.2.3. PCA Statistical Analyses of the Volatile Compounds

**Figure 7:**
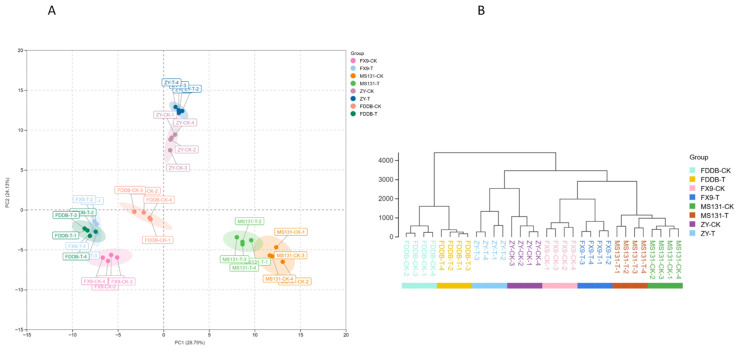
Principal component analysis (PCA) of the VOCs among tea samples (**A**) and clustering map (**B**).

To explore the aromatic differences among tea samples, principal component analysis (PCA) of the volatile compounds was conducted using statistical methods. The variance contribution rates for the two groups of principal components were 28.79% and 24.13% (Figure 7A), resulting in a cumulative variance contribution rate of 52.92%. The results (Figure 7A) showed that eight groups (FX9-CK, FX9-T, MS131-CK, MS131-T, ZY-CK, ZY-T, FDDB-CK, FDDB-T) can be clearly divided into eight regions. This suggests that the differences in VOCs have a marked influence on the tea samples from different withering methods, as evidenced by the principal component analysis. HCA was then conducted based on the identified volatile components, resulting in a heat map (Figure 7B) where rows represent volatile components and columns represent the tea samples. The HCA results clearly distinguished the eight tea samples.

### 3.3. Statistically Analyze the Degree of Change

**Figure 8:**
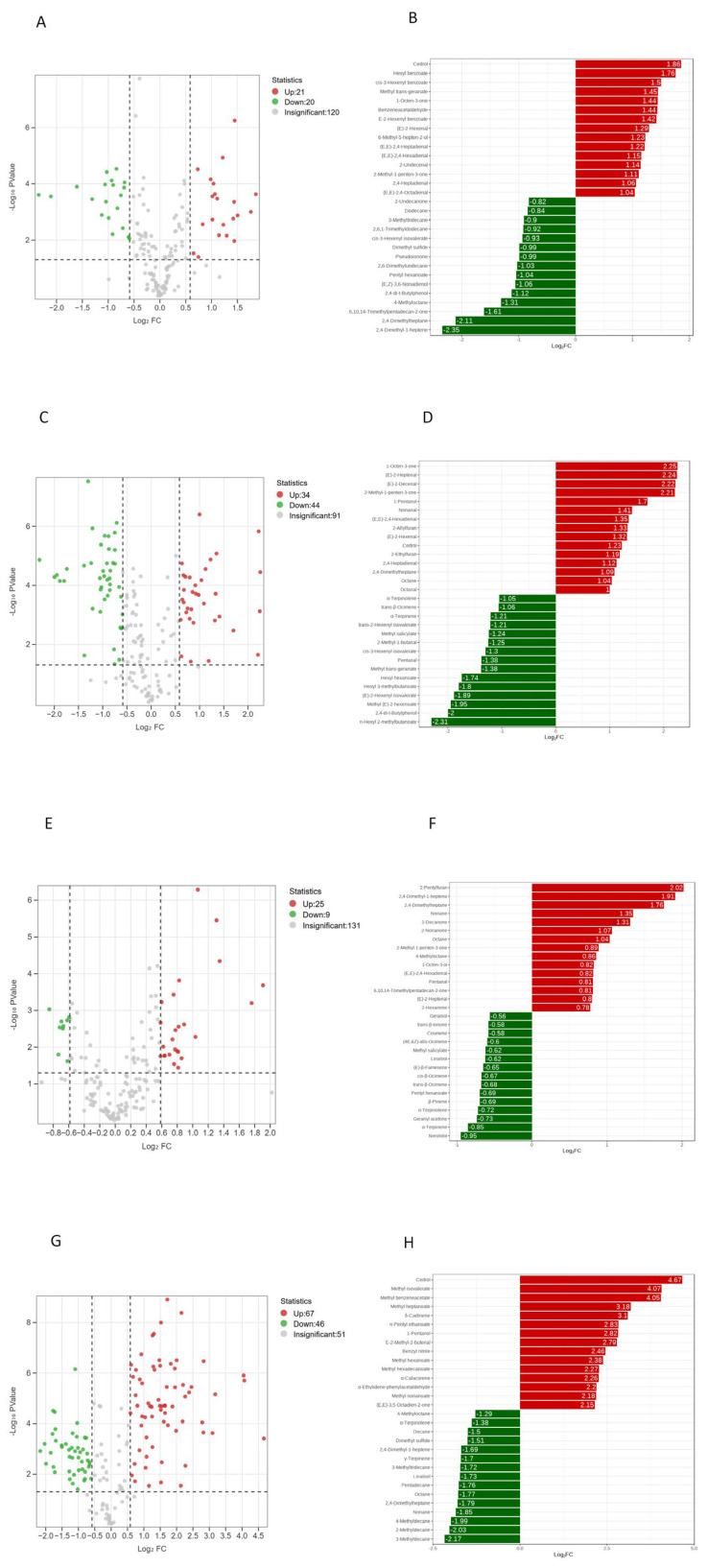
(**A**) Volcano plot showing the differential volatiles between MS131-CK and MS131-T. (**B**) Bar chart showing the top 30 significantly differential volatiles between MS131-CK and MS131-T. (**C**) Volcano plot showing the differential volatiles between FX9-CK and FX9-T. (**D**) Bar chart showing the top 30 significantly differential volatiles between FX9-CK and FX9-T. (**E**) Volcano plot showing the differential volatiles between ZY-CK and ZY-T. (**F**) Bar chart showing the top 30 significantly differential volatiles between ZY-CK and ZY-T. (**G**) Volcano plot showing the differential volatiles between FDDB-CK and FDDB-T. (**H**) Bar chart showing the top 30 significantly differential volatiles between FDDB-CK and FDDB-T.

Through temperature-changing withering technology, the aroma sensory quality of the four varieties of white teas was improved, and the characteristics of sweet and floral aroma were obvious. As depicted in Figure 7, key differential metabolites contributing to different withering were identified based on multivariate statistical analysis, using *p* < 0.05, and fold change ≥ 1.50 (upregulated) or ≤0.67 (down-regulated). In addition to the compounds mentioned above, the top 15 volatile compounds with the greatest change in content were made into a histogram (Figure 8).

The results in Figure 8A demonstrated that there was a total of 78 differential (34 upregulated and 44 down-regulated) volatile compounds between FX9-CK and FX9-T. In the comparison of FX9-T_vs_FX9-CK, according to the fold change value, the most down-regulated volatile compounds were n-hexyl-2-methylbutanoate, 2,4-di-t-Butylphenol, methyl (*E*)-2-hexenoate, (*E*)-2-hexenyl isovalerate, hexyl 3-methylbutanoate, hexyl hexanoate, methyl trans-geranate, cis-3-hexenyl isovalerate, methyl salicylate, trans-2-hexenyl isovalerate, and so on. The above compounds belonged to ester compounds, which exhibit fruity aroma characteristics. For instance, cis-3-hexenyl valerate has previously been identified as a key volatile compound in black tea from the ‘Baiye 1’ cultivar [2]. On the contrary, the most upregulated volatile compounds of FX9 samples were 1-octen-3-one, (*E*)-2-heptenal, (*E*)-2-decenal, 2-methyl-1-penten-3-one, 1-pentanol, nonanal, (*E*, *E*)-2,4-hexadienal, 2-allylfuran, (*E*)-2-hexenal, cedrol, 2,4-heptadienal, 2,4-dimethylheptane, octane, octanal, and so on. Among above compounds, decanal, (*E*, *E*)-2,4-nonadienal, (*E*)-2-decenal, nonanal, (*E*, *E*)-2,4-octadienal, 2,4-heptadienal, octanal, (*E*, *E*)-2,4-heptadienal, (*Z*)-4-hepten-1-al, heptanal, (*E*, *E*)-2,4-hexadienal, (*E*)-2-hexenal belonged to aldehydes, mainly presenting the characteristics of fresh and floral in white tea [38], and the increase in content is conducive to improving the quality of white tea.

Notably, the contents of 2-undecenal, cedrol, 1-octanol, and 1-octen-3-ol in white teas have increased, which are highly beneficial for enhancing the aroma quality of white tea, as these components contribute to the formation of floral fragrance in green tea, black tea, and white teas. Furthermore, the content of 2-allylfuran (Log2FC = 1.33) has increased after temperature-changing withering, which was also a substance with baking aroma. This phenomenon is consistent with the previously reported tea drying process.

The results in Figure 8C demonstrated that there was a total of 41 differential (21 upregulated and 20 down-regulated) volatiles between MS131-CK and MS131-T. In the comparison of MS131-T_vs_MS131-CK, according to the fold change value, the most down-regulated volatile compounds were 2,4-dimethyl-1-heptene, 2,4-dimethylheptane, 6,10,14-trimethylpentadecan-2-one, 4-methyloctane, 2,4-di-t-butylphenol, (*E*,*Z*)-3,6-nonadienol, pentyl hexanoate, 2,6-dimethylundecane, pseudoionone, cis-3-hexenyl isovalerate, 2,6,1-trimethyldodecane, 3-methyltridecane, dodecane, 2-undecanone, tetradecane, geranyl acetone. On the contrary, the most upregulated volatile compounds were cedrol, hexyl benzoate, cis-3-hexenyl benzoate, methyl trans-geranate, 1-octen-3-one, benzeneacetaldehyde, *E*-2-hexenyl benzoate, (*E*)-2-hexenal, 6-methyl-5-hepten-2-ol, (*E*,*E*)-2,4-heptadienal, 2-undecenal, 2-methyl-1-penten-3-one, 2,4-heptadienal, (*E*,*E*)-2,4-octadienal, hexanal, and heptanal. The above-mentioned volatile compounds, which belong to ketones, esters, and aldehydes, are common fragrance-producing substances in tea, which contribute to the flavor improvement of white tea.

The results in Figure 8E demonstrated that there was a total of 34 differential (25 upregulated and 9 downregulated) volatiles between ZY-CK and ZY-T. In the comparison of ZY-T_vs_ZY-CK, according to the fold change value, the most down-regulated volatile compounds were α-terpinene, geranyl acetone, α-terpinolene, myrcene, trans-*β*-ocimene, cis-*β*-ocimene, linalool, methyl salicylate, (4*E*,6*Z*)-allo-ocimene, which mostly belonged to olefins. On the contrary, the most up regulated volatile compounds were 2,4-dimethyl-1-heptene, 2,4-dimethylheptane, nonane, 2-decanone, 2-nonanone, octane, 2-methyl-1-penten-3-one, 4-methyloctane, 1-octen-3-ol, (*E*, *E*)-2,4-hexadienal, pentanal, 6,10,14-trimethylpentadecan-2-one, (*E*)-2-heptenal, 2-hexanone, 2-ethylfuran, 2-allylfuran, (*E*)-2-Decenal, 1-octen-3-one, 2,6,1-trimethyldodecane, dodecane, *E*-2-Methyl-2-butenal, 2,6-dimethylundecane, 2-undecanone, 3-octen-2-one, 1-octanol.

The results in Figure 8G demonstrated that there was a total of 113 differential (67 upregulated and 46 downregulated) volatiles between FDDB-CK and FDDB-T. In the comparison of FDDB-T_vs_FDDB-CK, according to the fold change value, the most down-regulated volatile compounds were 3-methyldecane, 2-methyldecane, 4-methyldecane, nonane, 2,4-dimethylheptane, octane, and pentadecane. The above compounds are all alkane substances, which are tasteless themselves and are not the key substances for the fragrance of tea, and the decrease in content has an effect on the fragrance quality of tea. Among the most upregulated volatile compounds, cedrol increased the most, with an FC value of up to 25.45, followed by methyl isovalerate, methyl benzeneacetate, methyl heptanoate, *δ*-Cadinene, n-pentyl ethanoate, 1-pentanol, *E*-2-methyl-2-butenal, benzyl nitrile, methyl hexanoate, methyl hexadecanoate, *α*-calacorene, *α*-ethylidene-phenylacetaldehyde, and methyl nonanoate. Particularly, the increasing contents of methyl isovalerate, methyl benzeneacetate, methyl heptanoate, methyl hexanoate, methyl hexadecanoate, and methyl nonanoate, which belonged to ester compounds, were helpful to the presentation of flower and fruit aroma of white tea samples.

Based on the above analysis, only the contents of 1-octen-3-one were significantly increased in all white teas of the four varieties (FX9, MS131, ZY, and FDDB) after temperature-changing withering. And the fold change values in the contents of 1-octen-3-one were 1.68 (FDDB), 4.77 (FX9), 2.71 (MS131), and 1.67 (ZY) after temperature-changing withering, respectively. And the contents of (*E*)-2-hexenal, 2,4-heptadienal, (*E*, *E*)-2,4-heptadienal, (*E*, *E*)-2,4-octadienal, which are primarily derived from fatty acid degradation, have increased significantly in white teas of the same three varieties (FDDB-T, FX9-T, MS131-T), after temperature-changing withering. Except for FDDB-T white teas, the contents of 2-methyl-1-penten-3-one have increased significantly in white tea of three varieties (FX9-T, MS131-T, ZY-T). Except for MS131-T white teas, the contents of (*E*, *E*)-2,4-hexadienal, (*E*)-2-heptenal, 1-octen-3-ol, 3-octen-2-one, and (*E*)-2-decenal have increased significantly in white teas of three varieties (FX9-T, FDDB-T, ZY-T). The above volatile compounds are basically present with a fresh, floral, and fruity fragrance, promoting the flavor formation in white teas.

Oppositely, only the contents of geranyl acetone have decreased significantly in all white teas of the four varieties (FX9, MS131, ZY, FDDB) after temperature-changing withering. According to reports, geranyl acetone presented a magnolia aroma with higher pungency, which reduced overall pleasantness [39]. So, the decreasing contents of geranyl acetone can improve the pleasantness of white tea samples. Except for MS131 white teas, the contents of methyl salicylate, myrcene, α-terpinene, *α*-terpinolene, *cis-β*-ocimene, trans-*β*-ocimene, and linalool have decreased significantly (FDDB-T, FX9-T, ZY-T), which is consistent with the literature that the heating process will reduce the contents of ocimene [40] and linalool [41] in teas. Except for Ziyan white teas, the contents of *cis*-3-hexenyl isovalerate have decreased significantly (FDDB-T, FX9-T, MS131-T). In this test, the decrease in the contents of *cis*-3-hexenyl isovalerate, a compound with a grassy aroma characteristic, was beneficial for improving the quality of white teas after temperature-changing withering.

### 3.4. Key Aroma-Active Volatiles Based on OAV Analysis

Only a few volatiles are thought to be important fragrance compounds that have a major impact on the overall olfactory profile, despite the fact that many volatiles have been identified in a variety of meals [35]. The flavor and qualitative attributes of tea are greatly influenced by the quantitative abundance and threshold of aroma-related chemicals in tea soup [35,42]. The contribution of fragrance compounds to the scent of tea samples was evaluated using OAVs; aroma compounds with an OAV > 1 were regarded as important taste components [43]. In this study, out of 176 identified volatiles, 76 exhibited odor activity value (OAV) greater than 1, and there were 68, 68, 71, 71, 71, 71, 69, 69 volatile compounds with OAV > 1 in MS131-CK, MS131-T, FX9-CK, FX9-T, ZY-CK, ZY-T, FDDB-CK, and FDDB-T, respectively (Table S1).

Although OAV is a widely used tool for screening potential aroma-active compounds, it has inherent limitations. For instance, it does not account for matrix effects, synergistic interactions, or inhibitory effects within the complex tea infusion [44]. Therefore, additional parameters were employed to comprehensively screen for characteristic aroma compounds.

To examine the effects of different methods on samples, significantly differential volatile components were chosen using the principle of OAV > 1, *p* < 0.05, and fold change ≥ 1.5 (upregulated) or ≤0.67 (down-regulated) at four varieties (Table S2). In the comparison of FDDB-T_vs_FDDB-CK, the contents of 24 differential volatile components have significantly increased (fold change ≥ 1.5, OAV > 1, *p* < 0.05), including cedrol, δ-Cadinene, methyl hexanoate, (*E*,*E*)-3,5-octadien-2-one, 2-hexanone, 6-methyl-5-heptene-2-one, trans-rose oxide, benzyl alcohol, (*E*,*E*)-2,4-hexadienal, (*E*,*E*)-2,4-heptadienal, 1-octen-3-ol, 3,5-octadien-2-one, (*E*,*E*)-2,4-nonadienal, 6-methyl-6-hepten-2-one, 2,4-decadienal, (*E*)-2-decenal, 1-heptanol, 2-nonanone, benzeneacetaldehyde, benzaldehyde, 1-octen-3-one, (*E*)-2-hexenal, *β*-damascenone, *β*-cyclocitral. Among the above volatile components, *β*-damascenone (apple-like) had the highest OAV, which were 7327.852 in FDDB-CK, and 11,180.221 in FDDB-T. In addition to improving aroma quality [45], *β*-damascenone also had higher sweetness similarity and significantly increased the sweet intensity of sucrose (*p* < 0.05) [46]. The OAVs of (*E*,*E*)-2,4-heptadienal were 2622.651 in FDDB-CK and 7539.865 in FDDB-T, which indicated (*E*,*E*)-2,4-heptadienal has a significant impact on the aroma quality of samples. (*E*,*E*)-2,4-heptadienal was identified as a key aroma substance in various Chinese teas, rapeseed oil, and various fruits, which contributes to fresh and floral aroma. After temperature-changing withering, the OAV of (*E*,*E*)-3,5-octadien-2-one in FDDB-T was greater than 1000, which correlated significantly with fruity aroma [47]. Benzaldehyde, which is associated with an almond-like fragrance, contributes to balancing the overall aroma profile of black and green teas [48]. The precursor of benzaldehyde exists in tea in the form of mandelonitrile glucoside, which hydrolyzes to produce mandelonitrile and then undergoes isomerization to yield benzaldehyde [49]. Benzeneacetaldehyde, showing an obvious sweet and honey aroma [42], is catalyzed by Amine oxidase (AO). It has been found that high temperature treatment during the withering process can significantly upregulate the expression of AO1, maoI (a copper amine oxidase gene) and At1g62810 (a copper/topa quinone amine oxidase gene), and promote the metabolism of phenylalanine to phenylacetaldehyde and phenylethanol during the withering process of white tea, which is consistent with the results of this study [50]. It is evident that temperature-changing withering technology significantly affects the OAV of volatile components. Particularly, the OAVs of cedrol have increased by 8 times from 0.318 in FDDB-CK to 8.086 in FDDB-T, which increases multiple times among all differential volatile components. Cedrol is a terpene alcohol widely existing in plants, described as a wood flavor, which is an important contributor to the wood flavor in many kinds of tea [51]. This increase is likely due to moderate cellular damage induced temperature-changing withering, which enhances enzyme–substrate interactions, accelerating protein degradation and sugar-amino acid conversion [52]. However, this proposed mechanism remains speculative and requires further experimental validation. On the contrary, the contents of 16 differential volatile components have significantly decreased (fold change ≤ 0.67, OAV > 1, *p* < 0.05) after temperature-changing withering, including linalool, dimethyl sulfide, decane, 2-pentylfuran, limonene, *trans*-*β*-ocimene, dimethyl trisulfide, methyl salicylate, decanal, *cis*-2-(2-Pentenyl) furan, *cis-β*-ocimene. The above volatile components were mostly positively correlated with the floral aroma, and showed temperature-changing withering may dilute the floral fragrance of the tea samples.

In the comparison of FX9-T_vs_FX9-CK, the contents of 17 differential volatile components have significantly increased (fold change ≥ 1.5, OAV > 1, *p* < 0.05), including 1-octen-3-one, (*E*)-2-decenal, nonanal, (*E*,*E*)-2,4-hexadienal, (*E*)-2-hexenal, cedrol, 2,4-heptadienal, 2-undecenal, trans-rose oxide, heptanal, (*E*,*E*)-2,4-heptadienal, decanal, (*E*,*E*)-2,4-nonadienal, 2,4-decadienal, naphthalene, 1-octen-3-ol. The above volatile components were mostly positively correlated with the fresh and floral aroma of teas. Among the above volatile components, (*E*,*E*)-2,4-heptadienal (fruit aroma) had the highest OAV, which was 5868.179 in FX9-CK and 9591.375 in FX9-T. Secondly, the OAVs of 1-octen-3-one increased from 1468.847 to 7011.019. As the differential volatile component, the OAVs of naphthalene (pungent aroma) have increased after temperature-changing, withering only in samples of FX9. On the contrary, the contents of 12 differential volatile components have significantly decreased (fold change ≤ 0.67, OAV > 1, *p* < 0.05) after temperature-changing withering, including hexyl 3-methylbutanoate, methyl salicylate, *trans*-*β*-ocimene, *cis*-*β*-ocimene, geranyl acetate, *δ*-cadinene, geraniol, myrcene, linalool, geranyl acetone, 6-methyl-5-heptene-2-one, and 2-undecanone. According to the literature report [53], *β*-ocimene (herbal) was positively correlated with the fresh aroma, and myrcene was positively correlated with fatty aroma. Their decreased contents had highlighted the fruitiness and floral aroma of the tea samples.

In the comparison of MS131-T_vs_MS131-CK, the contents of 10 differential volatile components have significantly increased (fold change ≥ 1.5, OAV > 1, *p* < 0.05), including cedrol, 1-octen-3-one, benzeneacetaldehyde, (*E*)-2-hexenal, (*E*, *E*)-2,4-heptadienal, 2-undecenal, hexanal, heptanal, *α*-pinene, cis-linalol oxide (pyranoid). On the contrary, the contents of seven differential volatile components have significantly decreased (fold change ≤ 0.67, OAV > 1, *p* < 0.05) after temperature-changing withering, including 2-(*E*,*Z*)-3,6-nonadienol, dodecane, 2-undecanone, geranyl acetone, hexyl 3-methylbutanoate, 2-decanone, and 5,6-epoxy-*β*-ionone. In the comparison of ZY-T_vs_ZY-CK, the contents of 10 differential volatile components have significantly increased (fold change ≥ 1.5, OAV > 1, *p* < 0.05), including 2-decanone, 2-nonanone, 1-octen-3-ol, (*E*,*E*)-2,4-hexadienal, 2-hexanone, (*E*)-2-decenal,1-octen-3-one, dodecane, 2-undecanone, and 1-octanol. On the contrary, the contents of five differential volatile components have significantly decreased (fold change ≤ 0.67, OAV > 1, *p* < 0.05) after temperature-changing withering, including myrcene, *trans*-*β*-ocimene, *cis*-*β*-ocimene, linalool, and methyl salicylate.

### 3.5. The Common Key Aroma Volatiles in Four Tea Varieties

**Figure 9:**
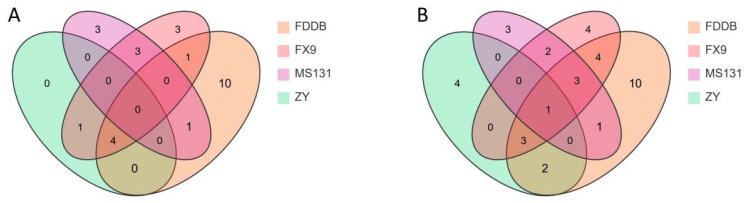
Venn diagram showing the number of down-regulated common key volatile compounds (**A**) and up-regulated common key volatile compounds (**B**) in four varieties.

**Figure 10:**
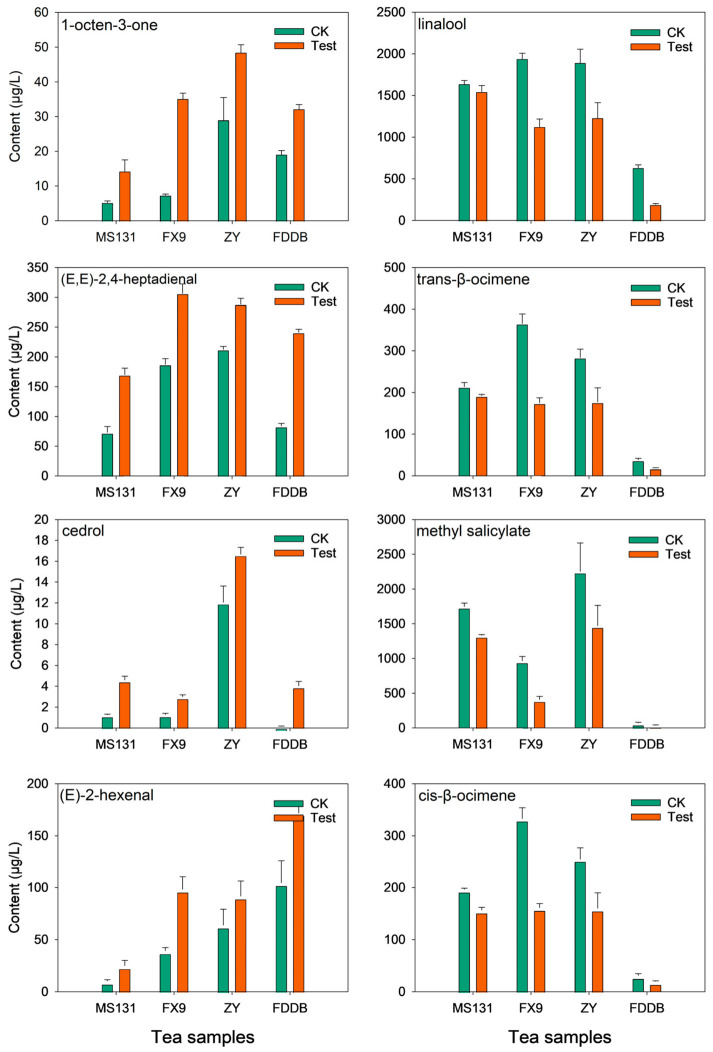
The common key volatile compounds in white teas under two withering treatments.

Based on the screening criteria of OAV > 1, *p* < 0.05, and fold change ≥ 1.5 or ≤0.67 (Table S2), the key aroma compounds identified across the four tea cultivars were subjected to Venn diagram analysis (Figure 9). The common key compounds among different tea cultivars are likely the causative factors underlying the quality changes induced by the novel processing technology. As in Figure 10, content variations in eight compounds were analyzed, including 1-octen-3-one, cedrol, (*E*,*E*)-2,4-heptadienal, (*E*)-2-hexenal, linalool, *trans-β*-ocimene, methyl salicylate, and *cis-β*-ocimene.

In four varieties (FDDB, FX9, MS131, ZY), there were no common volatile compound contents significantly decreased after temperature-changing withering. In FDDB, FX9, ZY, the contents of linalool, *trans*-*β*-ocimene, methyl salicylate, and *cis-β*-ocimene significantly decreased after temperature-changing withering. Linalool and *β*-ocimene are monoterpenoids released from glycosidic precursors (e.g., *β*-glucosides and *β*-primverosides) upon hydrolysis. Their anabolism and catabolism are influenced by tea processing techniques [54]. The reduction in contents of linalool, *trans-β*-ocimene, and *cis-β*-ocimene had a negative effect on the perception of floral and sweet aroma in white teas. While methyl salicylate had a strong wintergreen scent with higher pungency, which decreased overall pleasantness [55], decreasing its contents can improve the pleasantness of white tea samples.

In ZY and FX9, the contents of myrcene significantly decreased after temperature-changing withering, which imparted pine notes in teas. In FX9 and MS131, the contents of hexyl 3-methylbutanoate, geranyl acetone, and 2-undecanone significantly decreased after temperature-changing withering. In FDDB and FX9, only the contents of geraniol significantly decreased after temperature-changing withering, which presented floral freshness in teas. Geraniol, a representative glycosidically bound volatile, is released from its glycosidically bound form under heat or enzymatic hydrolysis. In this test, the contents of linalool have reduced in FDDB-T and FX9-T, indicating that temperature-changing withering causes its decomposition to exceed its synthesis. In FDDB and MS131, only the contents of dodecane significantly decreased after temperature-changing withering. However, dodecane with low concentration has almost no discernible odor, and the reduction in contents does not affect the aroma quality of white tea.

The common compounds exhibiting reduced contents, as mentioned above, originate from distinct biosynthetic pathways. Linalool and geraniol are generated via the hydrolysis of glycoside-bound precursors, whereas geranyl acetone is derived from the carotenoid degradation pathway. *Trans*-*β*-ocimene, *cis-β*-ocimene, and myrcene are synthesized within the chloroplasts of tea plants through the methylerythritol phosphate (MEP) pathway, with the entire biosynthetic process confined to the chloroplasts. Consequently, the regulatory pattern underlying the reduction in aroma compounds induced by the novel withering process remains unclear. The proposed pathways (glycoside hydrolysis, MEP pathway, and carotenoid degradation) are inferred from known biosynthetic routes [40,41,54] but have not been directly verified in this study.

In all white teas of the four varieties (FDDB, FX9, MS131, ZY), only the contents of 1-octen-3-one significantly increased after temperature-changing withering (Figure 10), and the OAVs of 1-octen-3-one were greater than 1000 in all samples. According to the literature, the odor threshold of 1-octen-3-one is only 0.005, which presents an obvious mushroom flavor, and it is an important aroma substance of green tea, black tea, and white tea [56]. The increase in the contents of 1-octen-3-one has contributed to the formation of the floral aroma in tea, which is consistent with the research findings on *Chloranthus* spicatus scented teas [57]. So temperature-changing withering (short-time high temperature) technology has significantly improved the aroma of white tea samples. The sensory-QDA and OAV results were further integrated to establish VOC-sensory linkages. The significant increase in 1-octen-3-one (OAV > 1000) and (*E*,*E*)-2,4-heptadienal (OAV 2622-9591) across cultivars corresponded to the enhanced fresh and floral notes in T samples (Figure 3). *β*-Damascenone (OAV 7327-11180), with its apple-like sweetness, directly contributed to the elevated sweet and fruity scores. Conversely, the reduction in linalool, trans-*β*-ocimene, and cis-*β*-ocimene-compounds associated with grassy and herbal notes aligned with the suppression of grassy aroma and improved overall pleasantness in temperature-changing withered teas.

In FDDB, FX9, MS131, the contents of cedrol, (*E*,*E*)-2,4-heptadienal, (*E*)-2-hexenal (Figure 10), significantly increased after temperature-changing withering. According to reports, cedrol, with a stale and woody aroma [50], had an odor threshold of 0.5, which was a characteristic aroma substance in white Peony teas and other teas [55,58]. Except for Ziyan white teas, the fold change values in the contents of cedrol were 25.45, 2.34, and 3.63, in FDDB, FX9, and MS131 samples, respectively, suggesting that temperature-changing withering can form the woody aroma of white teas. Cedrol was found in Tuo tea, Pu’er tea, and vintage white tea, and its content was positively correlated with the storage year, showing a stale aroma [59]. Previous studies have documented that thermal treatments—specifically the high-temperature drying employed in Lu’an Guapian green tea manufacture and extended roasting at 150 °C using rapeseed oil—result in marked increases in (*E*, *E*)-2,4-heptadienal content [29,60]. Such observations demonstrate that exposure to elevated processing temperatures enhances the formation of this volatile compound across tea products and various food systems.

In FDDB, FX9, ZY, the contents of (*E*,*E*)-2,4-hexadienal, 1-octen-3-ol, (*E*)-2-decenal, significantly increased after temperature-changing withering (Figure 10). As we know, 1-octen-3-ol, imparting mushroom notes, has a strong positive correlation with aroma in green tea [61], dark tea [62], white tea [63]. The literature demonstrated that drying temperature influenced the contents of 1-octen-3-ol in *Chloranthus* spicatus and aroma profiles of its scented green/black teas [57]. In MS131, FX9, and ZY, there were no common volatile compound contents significantly increased after temperature-changing withering. Regarding the common volatile compounds with increased contents, except for cedrol (a sesquiterpene), the formation of the other compounds, including 1-octen-3-one, (*E*,*E*)-2,4-heptadienal, (*E*)-2-hexenal, (*E*,*E*)-2,4-hexadienal, 1-octen-3-ol, and (*E*)-2-decenal, originates from the fatty acid oxidative degradation pathway. The increase in the contents of these compounds may be potentially associated with enhanced lipoxygenase (LOX) activity and subsequent oxidative degradation of fatty acid substrates during temperature-changing withering [30,60], though direct enzymatic or gene-expression evidence is required to confirm this hypothesis.

Although the OAV approach identified 1-octen-3-one, cedrol, (*E*,*E*)-2,4-heptadienal, and (*E*)-2-hexenal as common key contributors, we recognize that OAV alone cannot fully confirm sensory importance in the complex tea matrix [3,34]. Previous studies have demonstrated that aroma recombination and omission tests are necessary to validate the actual sensory contributions of these volatiles [3,59]. Future work will incorporate these sensory-directed analytical strategies to complement the current OAV-based screening.

Ziyan is a tea cultivar characterized by purple-leaf coloration and distinguished by its exceptionally high anthocyanin content, which sets it apart from the other three tea cultivars. For the three tea cultivars (FDDB, FX9, and MS131), the temperature-changing withering technique significantly increased the contents of 1-octen-3-one, cedrol, (*E*,*E*)-2,4-heptadienal, and (*E*)-2-hexenal, thereby promoting the fresh flavor profile of white tea. Based on these findings, this approach can be extended to a broader range of tea cultivars, thus establishing a novel method for processing white teas from small- and medium-leaf tea varieties.

## 4. Conclusions

In this study, a volatilomic analysis was conducted to investigate the effects of temperature-changing withering on the sensory aroma profiles and volatile compound compositions of white teas produced from four tea cultivars. The sensory evaluations of all white teas made from four cultivars indicated that temperature-changing withering enhanced the development of sweet and fruity aromas while suppressing grassy notes. Through HS-SPME-GC-MS, a total of 176 volatile compounds were identified. Significant alterations in the volatile compounds of four cultivars were observed following temperature-changing withering, characterized notably by increased levels of ketones, esters, and alkenes (*p* < 0.05). Based on the criteria of odor activity value (OAV) > 1, *p* < 0.05, and fold change ≥ 1.5, or ≤0.67, the number of key volatile compounds identified was 17 for MS131, 29 for FX9, 15 for ZY, and 40 for FDDB, respectively. Among the four tea cultivars subjected to temperature-changing withering, the contents of four volatile compounds were commonly upregulated, namely 1-octen-3-one, cedrol, (*E*,*E*)-2,4-heptadienal, and (*E*)-2-hexenal. Notably, each of these compounds exhibited multidimensional sensory contributions, promoting the fresh flavor profile of white teas. Nevertheless, the optimal parameters of temperature-changing withering in this study, require further validation under diverse fresh leaf conditions, including variations in plucking tenderness, harvest season, and growing regions.

## Data Availability

The original contributions presented in this study are included in the article/Supplementary Materials. Further inquiries can be directed to the corresponding author. The datasets used and/or analyzed during the current study are available from the corresponding author upon reasonable request.

## Supplementary Materials

The following supporting information can be downloaded at: https://www.mdpi.com/article/10.3390/foods15122120/s1,Table S1:Key volatile compounds with OAV > 1 in all samples; Table S2: Summary of common key volatile compounds in four tea varieties.Table S3: The concentration of volatile compounds in all samples (μg·L^−1^).

## Abbreviations

The following abbreviations are used in this manuscript:

MS131	Mingshan No. 131
FX9	Fuxuan No. 9
ZY	Ziyan
FDDB	Fudingdabai
FX9-CK	CK samples of ‘Fuxuan No. 9’ tea cultivar
FX9-T	Test samples of ‘Fuxuan No. 9’ tea cultivar
MS131-CK	CK samples of ‘Mingshan No. 131’ tea cultivar
MS131-T	Test samples of ‘Mingshan No. 131’ tea cultivar
ZY-CK	CK samples of ‘Ziyan’ tea cultivar
ZY-T	Test samples of ‘Ziyan’ tea cultivar
FDDB-CK	CK samples of ‘Fudingdabai’ tea cultivar
FDDB-T	Test samples of ‘Fudingdabai’ tea cultivar
PCA	Principal component analysis
OPLS-DA	Orthogonal projections to latent structures—discriminant analysis
OAV	Odor activity values

## References

[1] Fang J., Sureda A., Silva A.S., Khan F., Xu S., Nabavi S.M. (2019). Trends of tea in cardiovascular health and disease: A critical review. Trends Food Sci. Technol..

[2] Chen X., Dai P., Zhu Y., Jin W., Xie Y., Yang Z., Wang Z., Jin X., Lei M., Shi L. (2025). A triple-helix white tea polysaccharide alleviates ulcerative colitis via the gut microbiota–metabolites–inflammation regulatory axis. Carbohydr. Polym..

[3] Feng Z., Li M., Li Y., Yin J., Wan X., Yang X. (2022). Characterization of the key aroma compounds in infusions of four white teas by the sensomics approach. Eur. Food Res. Technol..

[4] Feng J., Zhuang J., Chen Q., Lin H., Chu Q., Chen P., Wang F., Yu B., Hao Z. (2024). The effect of maturity of tea leaves and processing methods on the formation of milky flavor in white tea-a metabolomic study. Food Chem..

[5] Shen S., Zhang J., Sun H., Zu Z., Fu J., Fan R., Chen Q., Wang Y., Yue P., Ning J. (2023). Sensomics-assisted characterization of fungal-flowery aroma components in fermented tea using *Eurotium cristatum*. J. Agric. Food Chem..

[6] Zhu W., Zhou S., Guo H., Hu J., Cao Y., Xu Y., Lin X., Tian B., Fan F., Gong S. (2024). Golden-flower fungus (*Eurotiwm cristatum*) presents fungal flower aroma as well as accelerates the aging of white tea (Shoumei). Food Chem..

[7] Zheng Z., Wu W., Wang Z., Zhang P., Ma B., Huang Y., Sun W. (2025). Molecular sensory basis of the unique “cocoa aroma” in white tea: An integrated study combining HS-SPME-GC–MS, GC-O, molecular docking, and addition tests. Food Res. Int..

[8] Zhou S., Zhang J., Ma S., Ou C., Feng X., Pan Y., Gong S., Fan F., Chen P., Chu Q. (2023). Recent advances on white tea: Manufacturing, compositions, aging characteristics and bioactivities. Trends Food Sci. Technol..

[9] Tan J., Engelhardt U.H., Lin Z., Kaiser N., Maiwald B. (2017). Flavonoids, phenolic acids, alkaloids and theanine in different types of authentic Chinese white tea samples. J. Food Compos. Anal..

[10] Wang Y., Yasir M., Walayat N., Ahmed I.A.M., Zhang W., Zheng J., Su Z., Wei R. (2025). Exploring the impact of diverse combinations of withering methods and drying temperatures on the quality characteristics of white tea. Ind. Crops Prod..

[11] Tan J., Fang Z., Tian C., Zhou C., Zhang C., Jiang L., Zheng A., Yang N., Guo Y. (2025). Improving flavor of Wuyi rock tea processed from rain-soaked leaves by optimizing withering conditions. Food Chem..

[12] Wang Y., Zheng P.-C., Liu P.-P., Song X.-W., Guo F., Li Y.-Y., Ni D.-J., Jiang C.-J. (2019). Novel insight into the role of withering process in characteristic flavor formation of teas using transcriptome analysis and metabolite profiling. Food Chem..

[13] Feng J., Ye S., Wang J., Wu J., Zhao J., Tian W., Pan G., Yu B., Qiu D., Lin H. (2025). From water migration to aroma development: Revealing the influence of environmental airflow on the aroma of white tea during withering. Food Chem..

[14] Fan Y., Yu T., Wang W., Zuo Y., Wang J., Ning J. (2025). LED spectral tailoring directs flavor metabolites during white tea withering. LWT.

[15] Hua J., Yuan H., Jiang Y., Cheng G., Wang W., Liu P. (2014). Effect of withering light intensity on physical characteristics and respiration of tea fresh leaves. J. Tea Sci..

[16] Yu X., Li Y., He C., Zhou J., Chen Y., Yu Z., Wang P., Ni D. (2020). Nonvolatile metabolism in postharvest tea (*Camellia sinensis* L.) leaves: Effects of different withering treatments on nonvolatile metabolites, gene expression levels, and enzyme activity. Food Chem..

[17] You F.-N., Deng H.-L., Hu J., Yao Z.-L., Wu S.-Q., Qin Y.-J., Tang T.-H., Sun Y. (2020). Effects of LED light withering at different temperatures on expression of key genes in the upstream of MEP and formation of volatiles in Tieguanyin tea. J. Integr. Agric..

[18] Muthumani T., Kumar R.S. (2007). Studies on freeze-withering in black tea manufacturing. Food Chem..

[19] Xia E.-H., Zhang H.-B., Sheng J., Li K., Zhang Q.-J., Kim C., Zhang Y., Liu Y., Zhu T., Li W. (2017). The tea tree genome provides insights into tea flavor and independent evolution of caffeine biosynthesis. Mol. Plant.

[20] Jia X., Zhang Q., Chen M., Wang Y., Lin S., Pan Y., Cheng P., Li M., Zhang Y., Ye J. (2023). Analysis of the effect of different withering methods on tea quality based on transcriptomics and metabolomics. Front. Plant Sci..

[21] Fang X., Liu Y., Xiao J., Ma C., Huang Y. (2023). GC–MS and LC-MS/MS metabolomics revealed dynamic changes of volatile and non-volatile compounds during withering process of black tea. Food Chem..

[22] Zhang X., Ling Z., Hu W., Xiang C., Cui L., Xu W., Xiao W. (2024). Effects of Different Temperature Hot Air Withering on Withered Leaves and Tea Quality of Black Tea. J. Tea Sci..

[23] Wu H., Sheng C., Lu M., Ke H., Li T., Wei Y., Shen S., Yin X., Lu C., Wang Y. (2024). Identification of the causes of aroma differences in white tea under different withering methods by targeted metabolomics. Food Biosci..

[24] Xu Y., Chen X., Mei L., You H., Lin Z., Guo H., Chu Q., Gong S., Fan F.J.F.F. (2026). Unveiling the Impact of Withering Methods on Aroma Profile of White Peony Tea Through Integrating Metabonomics and Sensomics. Food Front..

[25] Lin Y., Huang Y., Zhou S., Li X., Tao Y., Pan Y., Feng X., Guo H., Chen P., Chu Q. (2024). A newly-discovered tea population variety processed Bai Mu Dan white tea: Flavor characteristics and chemical basis. Food Chem..

[26] Jin L., Lian X., Yang Z., Li T., Li Y., He Q., Li D. (2024). Differences in aroma of Chuanhong Congou black tea of different tea plant varieties based on HS-SPME-GC-MS analysis. Shipin Gongye Ke-Ji.

[27] Liu C., Ren M., Ning M., Liao Y., Du X., Qin L., Chen W., Liu X., Wu A., Feng D. (2025). Cultivar-dependent variation in metabolomic profiles and sensory characteristics of Zhuyeqing green tea (*Camellia sinensis*). Food Biosci..

[28] Lai Y.-S., Li S., Tang Q., Li H.-X., Chen S.-X., Li P.-W., Xu J.-Y., Xu Y., Guo X. (2016). The dark-purple tea cultivar ‘Ziyan’accumulates a large amount of delphinidin-related anthocyanins. J. Agric. Food Chem..

[29] Lin L., Li K., Hua Y., Liao S., Chen J., Tan L., Yang Y., Sun B., Tang Q., Xu W. (2024). Dynamic changes of anthocyanins during ‘Ziyan’tea wine processing. Food Chem. X.

[30] Yuan X., Xue R., Jiang H., Luo X., Huang H., Li P. (2024). Purification and components identification of ‘Ziyan’anthocyanins. J. Food Compos. Anal..

[31] (2018). Tea Sensory Evaluation Methods.

[32] (2013). Tea—Preparation of Ground Sample and Determination of Dry Matter Content.

[33] Xu H., Pan S., Wang J., Ye T., Yan M., Liang X., Qian G., Yan T., Xin G. (2024). Comparative characterization of volatile organic compounds and aroma profiles in 10 Actinidia arguta cultivars by gas chromatography-mass spectrometry (GC–MS), sensory analysis, and odor activity value (OAV) combined with chemometrics. J. Food Compos. Anal..

[34] Lin H., Ye S., Feng J., Wang J., Kong W., Wu J., Zhang F., Zhao J., Guo J., Chen K. (2025). Impact of compression methods on flavor profile of white tea: Integrated analysis of appearance, aroma, and taste. Food Chem. X.

[35] Zhai X., Zhang L., Granvogl M., Ho C.T., Wan X. (2022). Flavor of tea (*Camellia sinensis*): A review on odorants and analytical techniques. Compr. Rev. Food Sci. Food Saf..

[36] Yin X.-L., Fu W.-J., Chen Y., Zhou R.-F., Sun W., Ding B., Peng X.-T., Gu H.-W. (2022). GC-MS-based untargeted metabolomics reveals the key volatile organic compounds for discriminating grades of Yichang big-leaf green tea. LWT.

[37] Chen L., Shi Y., Sun J., Wang H., Wang Y., Shu Z., He W., Dong C., Xu P. (2025). Deciphering the flavor profile and seasonal variation of black tea processed from cultivar ‘Baiye 1’. Food Res. Int..

[38] Zhang J., Wang Z., Zhang L., Huang W., Lin F., Xiao C., Zheng Z., Huang Y., Sun W. (2025). Underlying characteristic aroma of white tea from diverse geographical origins and its prediction. J. Sci. Food Agric..

[39] Yao M., Lakey P.S., Shiraiwa M., Zhao B. (2022). Volatile products generated from reactions between ozone and human skin lipids: A modelling estimation. Build. Environ..

[40] Chen H., Zhang X., Jiang R., Ouyang J., Liu Q., Li J., Wen H., Li Q., Chen J., Xiong L. (2023). Characterization of aroma differences on three drying treatments in Rucheng Baimao (*Camellia pubescens*) white tea. LWT.

[41] Sun X., Wang C., Long X., Han S., Xu W., Chen T., Liu J., Wen B., Li M. (2025). The impact of the unique two-stage bunch-drying on the formation of the floral aroma of Niangniang tea. Food Chem. X.

[42] Ni H., Jiang Q., Lin Q., Ma Q., Wang L., Weng S., Huang G., Li L., Chen F. (2021). Enzymatic hydrolysis and auto-isomerization during β-glucosidase treatment improve the aroma of instant white tea infusion. Food Chem..

[43] Xu K., Tian C., Zhou C., Zhu C., Weng J., Sun Y., Lin Y., Lai Z., Guo Y. (2022). Non-targeted metabolomics analysis revealed the characteristic non-volatile and volatile metabolites in the Rougui Wuyi rock tea (*Camellia sinensis*) from different culturing regions. Foods.

[44] Mosaferi S., Jelley R.E., Fedrizzi B., Barker D. (2022). Synthesis of d6-deuterated analogues of aroma molecules-β-damascenone, β-damascone and safranal. Results Chem..

[45] Wei Y., Yu Y.-Y., Li Y.-C., Zhong X.-Y., Zou C., Ning J., Dong W.-J., Wu K., Xu Y.-Q. (2025). Aroma compounds with enhanced sweet perception in tea infusions: Screening, characterization, and sweetening mechanism. J. Adv. Res..

[46] Wu H., Cui Y., Chen Y., Ren Q., Ge Z., Wang X., Yang X., Meng J., Chen M., Liao Y. (2025). Characterization of key aroma components of low-caffeine, high-theobromine, and high-polyphenol Camellia yungkianensis ‘Rongjiangcha’tea. LWT.

[47] Wang Q., Jiang X., Qin D., Liu S., Li H., Fang K., Wang Q., Li B., Pan C., Chen D. (2020). Metabolic profiling of flavor compounds in black teas with almond odor during processing. Eur. Food Res. Technol..

[48] Yang Z., Baldermann S., Watanabe N. (2013). Recent studies of the volatile compounds in tea. Food Res. Int..

[49] Guo X., Schwab W., Ho C.-T., Song C., Wan X. (2022). Characterization of the aroma profiles of oolong tea made from three tea cultivars by both GC–MS and GC-IMS. Food Chem..

[50] Li Q., Li Y., Luo Y., Xiao L., Wang K., Huang J., Liu Z. (2020). Characterization of the key aroma compounds and microorganisms during the manufacturing process of Fu brick tea. LWT.

[51] Yu Z., Yang Z. (2020). Understanding different regulatory mechanisms of proteinaceous and non-proteinaceous amino acid formation in tea (*Camellia sinensis*) provides new insights into the safe and effective alteration of tea flavor and function. Crit. Rev. Food Sci. Nutr..

[52] Huang F., Wu H., Luo F., Wang Y., Ye Y., Gong Y., Ye X. (2025). Effect of harvest seasons on biochemical components and volatile compounds in white teas from two cultivars. Foods.

[53] Dong F., Lai H., Sun Q., Yu W., Zhang G., Du S., Zhuang S., Liu T., Li Y., Fan X. (2025). Dynamic changes in metabolic compounds and their effects on flavor quality of Fuliang black tea and green tea during processing. Food Biosci..

[54] Wu W., Zheng Z., Wang Z., He B., Du S., Zeng W., Sun W. (2025). Identification of key aroma compounds contributing to the pleasurable sensory experience of white Peony tea using GC–MS, computational modeling, and sensory evaluation. Food Res. Int..

[55] Flaig M., Qi S.C., Wei G., Yang X., Schieberle P. (2020). Characterisation of the key aroma compounds in a Longjing green tea infusion (*Camellia sinensis*) by the sensomics approach and their quantitative changes during processing of the tea leaves. Eur. Food Res. Technol..

[56] Liu Y., Zhang X., Han M., Zhou H., Wang H., Yang J., Xu Y., Lei P. (2025). Effects of drying methods on volatile profiles of Chloranthus spicatus and quality of its scented tea: Chemometric approaches. Food Chem. X.

[57] Zhu Z., Yu J., Chen Y., Wang W., Du S., Wang D., Xue J., Tao Y. (2025). Characterization of key aroma compounds contributing to tea-aroma in green tea-flavor liquor. J. Food Compos. Anal..

[58] Zhao Y., Xiao K., Chen Y., Ma M., Tan J., Liu Z., Wang K., Huang J., Zhu M. (2025). Aroma Evolution and Key Volatile Profiles of Aged Raw Pu-erh Tea: Insights from Volatilomics, Electronic Sensing, and Microbiomics. J. Food Compos. Anal..

[59] Li Y., Zhou J., Xu W., He C., Zhu J., Zhang D., Chen Y., Yu Z., Wan X., Ni D. (2024). Key aroma components in Lu’an guapian green tea with different aroma types from five tea tree varieties decoded by sensomics. Food Biosci..

[60] Zhang L., Chen J., Zhang J., Sagymbek A., Li Q., Gao Y., Du S., Yu X. (2022). Lipid oxidation in fragrant rapeseed oil: Impact of seed roasting on the generation of key volatile compounds. Food Chem. X.

[61] Liu C., Wang C., Wei M., Ning M., Xu Z., Chen S., Cui J., Song C., Tang Q. (2026). Influence of leaf tenderness on the aroma and taste characteristics of green tea from *Camellia sinensis* cv. Chuancha No. 2: Integrated sensory and chemical profiling. Food Chem. X.

[62] Yang C., Lu X., Ho C.-T., Ma J., Wang Y., Chen Y., Yi Y., Zhu M., Wang Y., Liu Z. (2025). Comparison of solid-state fermentation with different Bacillus species on the volatile organic compounds and non-volatile metabolites of dark teas. Food Res. Int..

[63] Wu H., Wang X., Kong X., Shan R., Chen C., Li Z., Yu W. (2026). Impact of Aspergillus cristatus on Microbial Community and Flavor Profile of Fermented White Tea. LWT.

